# Heterologous Production of 2,2′-Dihydroxy Derivatives of Astaxanthin and Adonirubin in *Escherichia coli* and Evaluation of Their Antioxidant Activity

**DOI:** 10.3390/antiox15030327

**Published:** 2026-03-05

**Authors:** Rika Sekine, Miho Takemura, Misato Nagamori, Norihiko Misawa, Kazutoshi Shindo

**Affiliations:** 1 Department of Food and Nutrition, Japan Women’s University, 2-8-1 Mejirodai, Bunkyo-ku, Tokyo 112-8681, Japan; m2002020sr@ug.jwu.ac.jp (R.S.); m2202021nm@ug.jwu.ac.jp (M.N.); 2 Research Institute for Bioresources and Biotechnology, Ishikawa Prefectural University, 1-308 Suematsu, Nonoichi-shi 921-8836, Japan; mtake@ishikawa-pu.ac.jp (M.T.); n-misawa@ishikawa-pu.ac.jp (N.M.)

**Keywords:** 2,2′-dihydroxyastaxanthin, 2,2′-dihydroxyadonirubin, *Escherichia coli*, antioxidant activity

## Abstract

Astaxanthin is a prominent carotenoid with strong antioxidant activity due to its 13 conjugated double bonds and its 3,3′-hydroxy groups adjacent to its 4,4′-carbonyl groups. This red pigment is utilized as a food additive and nutritional supplement, and it also has applications in cosmetics. But the extremely low water solubility of astaxanthin limits its broader commercial application. In order to decrease the hydrophobic property of astaxanthin, we produced 2,2′-dihydroxy derivatives of astaxanthin and its intermediate adonirubin, (2*R*,3*S*,2′*R*,3′*S*)-2,2′-dihydroxyastaxanthin (**1**) and (2*R,3S,*2′*R*)*-*2,2′-dihydoxyadonirubin (**2**), in the cells of *Escherichia coli* as dominant carotenoids. This result was achieved by using the *crtG* gene that codes for zeaxanthin/canthaxanthin/astaxanthin 2,2′-hydroxylase, derived from *Brevundimonas* sp. strain SD212, in addition to astaxanthin biosynthesis genes that carry the *Haematococcus pluvialis IDI*, *Pantoea ananatis crtE*, *crtB*, *crtI*, *crtY*, *crtZ*, and *Paracoccus* sp. N81106 *crtW* genes. The singlet oxygen-quenching activities of **1** and **2** (IC_50_ 4.3 μM and 8.3 μM, respectively) were examined and found to be comparable to that of astaxanthin (IC_50_ 1.7 μM).

## 1. Introduction

Carotenoids are synthesized in all photosynthetic organisms that include photosynthetic bacteria, cyanobacteria, algae, and plants, as well as in some non-photosynthetic microorganisms, which include bacteria and yeasts (fungi) [1,2,3]. These pigments are generally believed to protect their cells from (photo)oxidative stress, which is thought to be their main function [4,5]. Astaxanthin (3,3′-dihydroxy-β,β-carotene-4,4′-dione) is a prominent carotenoid with potent antioxidant properties due to its 13 conjugated double bonds between the 4,4′-carbonyl groups on its β rings, as well as its 3,3′-hydroxy groups being adjacent to its 4,4′-carbonyl carbons. Among the organisms that can de novo synthesize carotenoids (carotenogenic organisms), astaxanthin, which is typically of the 3*S*,3′*S*-form, can be produced in several microalgae such as *Haematococcus pluvialis* [6,7]; the *Adonis aestivalis* flower [8]; the Heterobasidiomycetous yeast *Xanthophyllomyces dendrorhous* (untypical 3*R*,3′*R*-form) [9]; and some species from the phylum Proteobacteria (recently renamed *Pseudomonadota*), e.g., the genera *Paracoccus* and *Brevundimonas* [10,11,12,13]. Astaxanthin synthesized in *H. pluvialis* and the Adonis flower are frequently present in esterified forms [8,14].

Astaxanthin and its esters are also found in some organisms that cannot de novo synthesize carotenoids, e.g., the shells and eggs of crustaceans such as crabs and shrimps and in the body surfaces or muscles of some fishes through their intake of astaxanthin-producing small crustaceans or through the metabolic conversion of dietary carotenoids such as β-carotene into astaxanthin [14].

Astaxanthin has been reported to exhibit various pharmaceutical activities, including protective effects against cardiovascular diseases [15,16], cancer [17], diabetes [18,19], neurodegenerative disorders, and immune dysfunction [20,21]. Owing to these properties, it is widely used in nutritional supplements, food products, and cosmetic formulations [22].

The extremely low water solubility of astaxanthin, resulting from its high hydrophobicity, limits its broader commercial application, and numerous strategies have been explored to improve its dispersibility in water. A marine bacterium *Agrobacterium aurantiacum*, later shown to belong to the genus *Paracoccus*., was found to produce glycosylated astaxanthin such as astaxanthin glucoside [23]. The heterologous production of glycosylated astaxanthin, typically its 3,3′-diglucoside, has been carried out with *E. coli* cells or other microbial hosts [24,25,26,27,28]. A water-soluble complex of astaxanthin was constructed using arabinogalactan [29]. The construction of PEGylated astaxanthin [30,31] and nanoencapsulated astaxanthin have been prepared [32], while these technologies still face several unresolved challenges in the manufacturing domain [32,33]. In this study, we aimed to generate structurally related compounds that exhibit high aqueous solubility, improved bioavailability, and pharmacological activities comparable to those of astaxanthin by introducing OH function at C-2 in the β-ionone ring. The *crtG* gene that codes for β,β-carotenoid (carotenoid including the two β-end groups) 2,2′-hydroxylase was discovered in a marine bacterium *Brevundimonas* sp. Strain, SD212, that can produce 2,2′-dihydroxyastaxanthin [11,12]. The *Brevundimonas* CrtG protein (2,2′-hydroxylase) was found to accept not β-carotene but zeaxanthin and canthaxanthin as the substrates to synthesize nostoxanthin and 2,2′-dihydroxycanthaxanhin, respectively [11]. It was also demonstrated that the CrtG enzyme converts astaxanthin into 2-hydroxyastaxanthin [11]. Since then, *crtG* or *crtG*-like genes with the same or similar functions to the SD212 *crtG* gene have been identified in various bacteria, e.g., a non-marine bacterium *Brevundimonas aurantiaca* [34] and the nostoxanthin-producing *Sphingomonas elodea* ATCC 31461 (class *Alphaproteobacteria*; phylum *Pseudomonadota*) [35], a marine bacterium *Algoriphagus* sp. oki45 (phylum *Bacteroidota*) [36], and a cyanobacterium, namely *Thermosynechococcus elongatus* strain BP-1 [37]. Although microbial production of 2,2′-dihydroxyastaxanthin and 2-hydroxyastaxanthin have been reported, including their production with native bacterial producers [11,12,34,38], few biological activities were present, most likely due to its limited production.

In this study, we were able to efficiently produce two highly polar astaxanthin-related carotenoids, which were identified as the 2,2′-dihydroxy derivatives of astaxanthin and its intermediate adonirubin, i.e., (2*R*,3*S*,2′*R*,3′*S*)-2,2′-dihydroxyastaxanthin (rare carotenoid) and (2*R*,3*S,*2′*R*)*-*2,2′-dihydoxyadonirubin (new carotenoid), respectively. This result was achieved by introducing the SD212 *crtG* and astaxanthin biosynthetic genes into *E. coli* cells as a heterologous host. We also evaluated the singlet oxygen (^1^O_2_)-quenching activities of both compounds.

## 2. Materials and Methods

### 2.1. Bacterial Strains

*E. coli* K12 DH5α was used for plasmid construction. *E. coli* K12 JM101(DE3) was used for expression of the carotenoid biosynthesis genes as described previously [39].

### 2.2. Construction of Plasmids Containing Carotenogenesis Genes for Expression in E. coli

**Figure 1:**
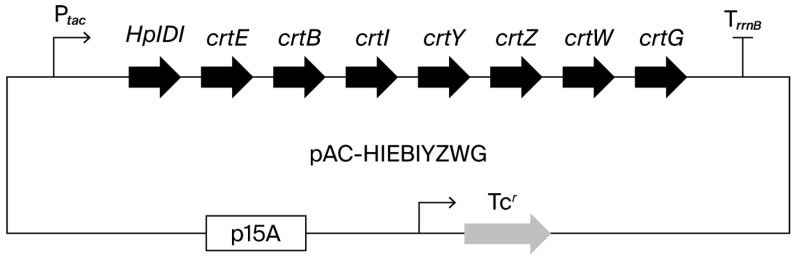
Plasmid constructs of pRK-HIEBIYZWG. *HpIDI*, the *H. pluvialis* isopentenyl diphosphate isomerase gene; *crtE*, the *P. ananatis* geranylgeranyl diphosphate synthase gene; *crtB*, the *P. ananatis* phytoene synthase gene; *crtI*, the *P. ananatis* phytoene desaturase gne; *crtY*, the *P. ananatis* lycopene β-cyclase gene; *crtZ*, the *P. ananatis* β-carotene/canthaxanthin 3,3′-hydroxylase gene; *crtW*, the *Paracoccus* sp. N81106 β-carotene/zeaxanthin 4,4′-ketolase gene; and *crtG*, the *Brevundimonas* sp. SD212 zeaxanthin/canthaxanthin/astaxanthin 2,2′-hydroxylase gene.

To construct a plasmid pRK-HIEBIYZWG (Genbank/DDBJ accession no. LC896679), the *crtG* gene of *Brevundimonas* sp. strain SDS212 was artificially synthesized to meet the *E. coli* codon usage. It was inserted between *crtW* and the *rrnB* terminator (T*_rrnB_*) of the plasmid pRK-HIEBIYZW (Genbank/DDBJ accession no. LC637573) [40]. The structure of this plasmid is shown in Figure 1.

### 2.3. Solvents and Reagents

Analytical-grade dichloromethane (CH_2_Cl_2_), *n*-hexane, acetone, methanol (MeOH), ethyl acetate (EtOAc), tetrahydrofuran (THF), and *tert*-buthyl methyl ether (*t*-BME) were obtained from Kanto Chemical Co., Inc. (Tokyo, Japan). All other reagents were purchased from Sigma-Aldrich (St. Louis, MO, USA).

### 2.4. Spectroscopic Analysis Using NMR and MS

NMR spectra [^1^H and ^13^C NMR, double quantum-filtered correlation spectroscopy (DQF COSY), heteronuclear single quantum correlation (HSQC), heteronuclear multiple bond coherence (HMBC), and nuclear Overhauser effect spectroscopy (NOESY)] were obtained via spectrometry (Bruker AVANCE400 spectrometer (Bruker BioSpin GmbH, Rheinstetten, Germany)) and were analyzed in TopSpin1.3. Chemical shifts were referenced to residual solvent signals (CDCl_3_: δ_H_ 7.26, δ_C_ 77.0). Electrospray ionization mass spectrometry (ESI-MS) was performed using a JEOL JMS-T100LP instrument.

### 2.5. Culture of E. coli Transformant

*E. coli* transformants were cultured in 100 mL 2 × YT (2 YT) medium (16 g/L of tryptone, 10 g/L of yeast extract, and 5 g/L of NaCl) supplemented with 10 mg/L of tetracycline in a 500 mL Sakaguchi flask at 37 °C at 150 rpm for 24 h (preculture) or at 22 °C at 150 rpm for 48 h (main culture).

### 2.6. Extraction and HPLC Analysis of Carotenoids from E. coli Cells

The cultured *E. coli* cells (100 mL) were centrifuged (7000× *g*, 15 min), and the precipitate was extracted with 5 mL of acetone–MeOH (7:2) (*v*/*v*) and 5 mL of CH_2_Cl_2_–MeOH (1:1) (*v*/*v*) with sonication in a stepwise manner. The mixed extract was centrifuged (7000× *g*, 5 min), and the supernatant (10 mL) was analyzed using octadecylsilyl silica gel (ODS) HPLC [HPLC conditions: column PEGASIL ODS SP100 (Senshu Scientific Co. Ltd., Tokyo, Japan) of i.d. 4.6 mm × 150 mm; solvent A: 50% (*v*/*v*) CH_3_CN, solvent B: CH_3_CN:*t*-BME (7:3) (*v*/*v*). 0→5 min A 100%, 5→20 min A 100%→B 100% linear gradient, 20→30 min B 100%; flow rate 1.0 mL/min; detection 450 nm].

### 2.7. Isolation of Carotenoids Produced by Cells Transfected with pRK-HIEBIYZWG

The transformed cells of *E. coli* JM101 (DE3) carrying pRK-HIEBIYZWG were collected from 10 L of culture by centrifugation (7000 × *g*, 15 min) and extracted using 100 mL of acetone–MeOH (7:2) (*v*/*v*) and 100 mL of CH_2_Cl_2_–MeOH (1:1) (*v*/*v*) with sonication in a stepwise manner. The mixed extract (200 mL) was concentrated to a small volume in vacuo and then partitioned with *n*-hexane and 90% methanol (*v*/*v*) (each 150 mL). The 90% (*v*/*v*) MeOH layer containing the carotenoids was concentrated to dryness to produce red oil (3.54 g). The red oil was partitioned with EtOAc and water (each 150 mL), and the EtOAc layer containing the carotenoids was concentrated to dryness to produce a red powder (276.0 mg). This red powder underwent silica gel column chromatography (i.d. 20 × 200 mm) (CHROMATOREX FL60D, Fuji silysia chemical Ltd., Aichi, Japan) in *n*-hexane/acetone (5:3) (*v*/*v*), and frs. 17-29 were collected and concentrated to dryness to produce crude carotenoids (42.7 mg). The crude carotenoids were subjected to preparative octadecylsilyl silica gel (ODS) high-performance chromatography (HPLC). The HPLC conditions were as follows: column, PEGASIL ODS SP100 (Senshu Scientific Co. Ltd., Tokyo, Japan) of i.d. 10 × 250 mm; solvent, 90% (*v*/*v*) MeOH; flow rate, 3.0 mL/min; and detector, photodiode array (PDA) (250-700 nm). In this chromatography, carotenoids **1** and **2** were eluted as pure compounds (**1**: *t_R_* 15.4 min (4.3 mg) and **2**: *t_R_* 22.2 min (12.0 mg)).

### 2.8. Singlet Oxygen-Quenching Activity

For the measurement of singlet oxygen-quenching activity, 160 μL of 12.5 μM methylene blue and 200 μL of 0.12 M linoleic acid, with or without 40 μL of carotenoid (final concentration of 1–25 μM, each dissolved in ethanol), were added to 5 mL glass test tubes. The solution was thoroughly mixed and illuminated at 7000 lx and 22 °C for 3 h in a Styrofoam box. Subsequently, 120 μL of the reaction mixture was removed and diluted to 3.48 mL with ethanol. A_235_ was measured to estimate the formation of conjugated dienes. The percentage quenching was calculated as follows: (1-[T-B]/C) × 100 (%), where T, C, and B are the A_235_ readings of the illuminated mixture containing carotenoid (T), the illuminated control (no carotenoid) (C), and the zero control (non-illuminated carotenoid) (B), respectively. Activity was expressed as the half-maximal inhibitory concentration (IC_50_). All experiments were done in triplicate.

### 2.9. Radical Scavenging Activity (DPPH) Assay

1,1-diphenyl-2-picryhydrazyl (DPPH) radical scavenging activity was determined according to the method of Liu et al. [38]. One mL of carotenoid MeOH solution with different concentrations (5, 10, 15, and 20 μg/mL) and 3 mL of DPPH methanol solution (0.1 mmol/L) were mixed. After 30 min reaction in the dark at room temperature, the absorbance (517 nm) of the mixture was then measured. The percentage scavenging was calculated as follows: (1 − [T − B]/C) × 100 (%), where T, C, and B are the OD_517_ readings of the mixture containing carotenoid and DPPH (T), the control (no carotenoid and DPPH) (C), and the zero control (carotenoid and no DPPH) (B), respectively. All experiments were done in triplicate.

### 2.10. Assigned ^1^H and ^13^C NMR Data of (2R,3S,2′R,3′S)-2,2′-Dihydroxyastaxanthin (***1***) and (2R,3S,2′R)-2,2′-Dihydoxyadonirubin (***2***)

(2*R*,3*S*,2′*R*,3′*S*)-2,2′-dihydroxyastaxanthin (**1**)

^1^H NMR (CDCl_3_) δ: 1.27 (6H, s, H-16 and H-16′), 1.30 (6H, s, H-17 and H-17′), 1.95 (6H, s, H-18 and H-18′), 1.99 (6H, s, H-20 and H-20′), 2.01 (6H, s, H-19 and H-19′), 3.54 (2H, d, *J* = 11.5 Hz, H-2 and H-2′), 4.17 (2H, d, *J* = 11.5 Hz, H-3 and H-3′), 6.25 (2H, d, *J* = 16.3 Hz, H-7 and H-7′), 6.30 (2H, d, *J* = 11.9 Hz, H-14 and H-14′), 6.31 (2H, d, *J* = 11.0 Hz, H-10 and H-10′), 6.44 (2H, d, *J* = 16.3 Hz, H-8 and H-8′), 6.45 (2H, d, *J* = 15.3 Hz, H-12 and H-12′), 6.66 (2H, dd, *J* = 11.0, 15.3 Hz, H-11 and H-11′), 6.68 (2H, m, H-15 and H-15′).

^13^C NMR (CDCl_3_) δ: 12.6 (C-19 and C-19′), 12.8 (C-20 and C-20′), 14.0 (C-18 and C-18′), 20.2 (C-16 and C-16′), 25.7 (C-17 and C-17′), 41.8 (C-1 and C-1′), 74.1 (C-3 and C-3′), 77.5 (C-2 and C-2′), 122.7 (C-7 and C-7), 124.6 (C-11 and C-11′), 126.9 (C-5 and C-5′), 130.7 (C-15 and C-15′), 133.9 (C-14 and C-14′), 134.5 (C-10 and C-10′), 135.4 (C-9 and C-9′), 136.7 (C-13 and C-13′), 139.9 (C-12 and C-12′), 142.7 (C-8 and C-8′), 162.1 (C-6 and C-6′), 198.2 (C-4 and C-4′).

(2*R*,3*S*,2′*R*)*-*2,2′-dihydoxyadonirubin (**2**)

^1^H NMR (CDCl_3_) δ: 1.21 (3H, s, H-16′), 1.25 (3H, s, H-17′), 1.27 (3H, s, H-16), 1.30 (3H, s, H-17), 1.89 (3H, s, H-18′), 1.95 (3H, s, H-18), 1.99 (6H, s, H-20 and H-20′), 2.01 (6H, s, H-19 and H-19′), 2.61 (1H, dd, *J* = 9.1, 17.1 Hz, H-3′a), 2.79 (1H, dd, *J* = 4.3, 17.1 Hz, H-3′b), 3.54 (1H, d, *J* = 11.5 Hz, H-2), 3.89 (1H, dd, *J* = 4.3, 9.1 Hz, H-2′), 4.17 (1H, d, *J* = 11.5 Hz, H-3), 6.23 (1H, d, *J* = 15.5 Hz, H-7′), 6.25 (1H, d, *J* = 16.3 Hz, H-7), 6.30 (2H, d, *J* = 11.0 Hz, H-14 and H-14′), 6.31 (2H, d, *J* = 11.0 Hz, H-10 and H-10′), 6.44 (1H, d, *J* = 16.3 Hz, H-8), 6.45 (2H, d, *J* = 15.3 Hz, H-12 and H-12′), 6.45 (1H, d, *J* = 15.5 Hz, H-8′), 6.66 (2H, dd, *J* = 11.0, 15.3 Hz, H-11 and H-11′), 6.68 (2H, m, H-15 and H-15′).

^13^C NMR (CDCl_3_) δ: 12.6 (C-19 and C-19′), 12.8 (C-20 and C-20′), 13.8 (C-18′), 14.0 (C-18), 20.2 (C-16), 21.5 (C-16′), 25.7 (C-17), 25.7 (C-17′), 41.3 (C-1′), 41.8 (C-1), 42.6 (C-3′), 74.1 (C-3), 74.2 (C-2′), 77.5 (C-2), 122.7 (C-7), 123.7 (C-7′), 124.6 (C-11), 124.7 (C-11′), 126.9 (C-5), 130.1 (C-5′), 130.6 (C-15′), 130.8 (C-15), 133.6 (C-14), 133.9 (C-14′), 134.4 (C-10′), 134.6 (C-10), 134.7 (C-9′), 135.4 (C-9), 136.6 (C-13′), 136.7 (C-13), 139.4 (C-12′), 139.9 (C-12), 141.6 (C-8′), 142.7 (C-8), 159.6 (C-6′), 162.1 (C-6), 197.0 (C-4′), 198.2 (C-4).

### 2.11. Statistical Analysis

Data obtained from the ^1^O_2_ quenching assay were analyzed using one-way analysis of variance among subjects, and post hoc comparisons were made using Student’s *t* test. In all cases, statistical significance was set at *p* < 0.05.

## 3. Results

**Figure 2:**
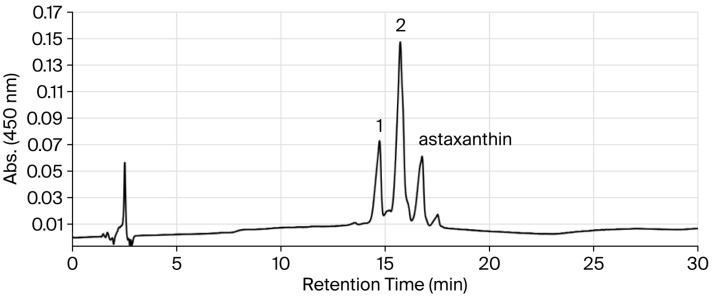
ODS HPLC analysis of carotenoids produced in *E. coli* (pRK-HIEBIYZWG).

Carotenoid pigments produced by recombinant cells of *E. coli* JM101 (DE3), which carried plasmid pRK-HIEBIYZWG, were analyzed using ODS HPLC. This *E. coli* transformant mainly produced two major carotenoids in addition to astaxanthin, as shown in Figure 2. The production ratio of **1**, **2**, and astaxanthin was approximately 1:3:1.

Carotenoids **1** and **2** were purified by extraction from the cells with organic solvents, two-layer partitioning, silica gel column chromatography, and preparative ODS HPLC. The purified carotenoids **1** and **2** were analyzed using ESI-MS (+), 1D (^1^H and ^13^C), and 2D (^1^H-^1^H DQF COSY, HSQC, HMBC, and NOESY) NMR spectra. The ^1^H and ^13^C NMR spectra of carotenoids **1** and **2** in CDCl_3_ are shown in Figures S1–S4.

**Figure 3:**
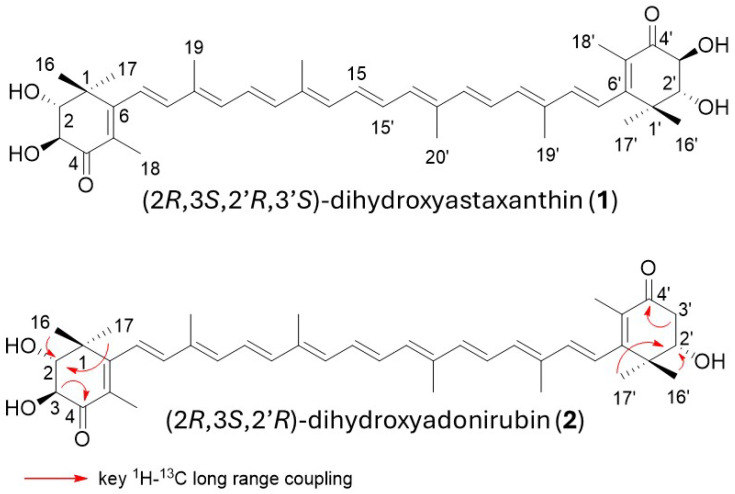
The structures of the produced carotenoids, and the key ^1^H-^13^C long-range couplings observed in the HMBC experiment.

Based on the results of ESI-MS and NMR analyses (Figures S1 and S2), **1** was unambiguously identified as (2*R*,3*S*,2′*R*,3′*S*)-2,2′-dihydroxyastaxanthin [semi-IUPAC name, (2*R*,3*S*,2′*R*,3′*S*)-2,3,2′,3′-tetrahydroxy-β,β-carotene-4,4′-dione], which can simply be called 2,2′-dihydroxyastaxanthin (Figure 3). The ^1^H and ^13^C NMR data of (2*R*,2′*R*)-2,2′-dihydroxyastaxanthin, which had been reported to be purified from the strain SD212 [12], showed very good agreement with those of **1**.

The molecular formula of **2** was determined as C_40_H_52_O_5_ using HR-ESI-MS analysis. Analyses of ^1^H and ^13^C NMR of **2** in CDCl_3_ (Figures S3 and S4) proved that **2** possesses a structure very similar to astaxanthin, while its structure is asymmetrical. From the overview of the ^1^H and ^13^C NMR spectra, **2** was confirmed to possess the same all-*trans* polyene chain linked to two 4-keto-β-ionone rings as astaxanthin. In the ^1^H NMR spectrum of **2**, three oxygen-attached sp3 methine signals (H-2 (δ 3.54, d, *J* = 11.5 Hz), H-2′ (δ 3.89, dd, *J* = 4.3, 9.1 Hz), and H-3 (δ 4.17, d, *J* = 11.5 Hz)) and one sp3 methylene signal (H-3′a (δ 2.61, dd, *J* = 9.1, 17.1 Hz) and H-3′b (δ 2.79, dd, *J* = 4.3, 17.1 Hz) were observed, and vicinal spin networks between methine H-2 and H-3 and between methine H-2′ and methylene H-3′ (H-3′a and H-3′b) were observed in the ^1^H-^1^H DQF COSY spectrum. After assigning all directly bonded ^1^H–^13^C correlations based on the HSQC spectrum, we proceed to analyze the HMBC spectrum. In the HMBC spectrum, ^1^H-^13^C long-range couplings were observed from H-16 (δ 1.29) and H-17 (δ 1.30) to C-2 (δ 77.5), from H-3 to C-4 (δ 198.2), from H-16′ (δ 1.21) and H-17′ (δ 1.25) to C-2′ (δ 74.2), and from H-3′a and H-3′b to C-4′ (δ 197.0) (Figure 3). From these findings and the stereoselectivities of the used 2- and 2′-OH-inducing biosynthetic genes (c*rtG*), the structure of **2** was determined as (2*R*,2′*R*,3*S*)-dihydroxyadonirubin [semi-IUPAC name, (2*R*,3*S*,2′*R*)-2,3,2′-trihydroxy-β,β-carotene-4,4′-dione], which can be called (2*R*,3*S*,2′*R*)-2,2′-dihydroxyadonirubin, or more simply, 2,2′-dihydroxyadonirubin (Figure 3). Compound **2** is a new carotenoid according to the CAS database. (2*R*,3*S*,2′*R*,3′*S*)-2,2′-dihydroxyastaxanthin (**1**), (2*R*,3*S*,2′*R*)-2,2′-dihydroxyadonirubin (**2**), and astaxanthin were all stable in solution throughout the isolation and structural elucidation process.

We examined the singlet oxygen (^1^O_2_)-quenching activities of **1**, **2**, and astaxanthin. The results are shown in Table 1 with those for canthaxanthin, which we evaluated previously [41]. The singlet oxygen-quenching activities of **1** and **2** were slightly lower than those of astaxanthin. We next examined the DPPH radical scavenging activities of **1**, **2**, and astaxanthin. None of the compounds showed significant scavenging activity at 5–20 μg/mL (Figure S5).

## 4. Discussion

Yokoyama et al. (1996) [12] reported that a marine bacterium strain, SD212, which was later shown to belong to the genus *Brevundimonas* (class *Alphaproteobacteria*) [11], synthesized the 2,2′-dihydroxy or 2-hydroxy derivatives of astaxanthin or its intermediates, i.e., (2*R*,3*S*,2′*R*,3′*S*)-2,2′-dihydroxyastaxanthin, (2*R*,3*S*,3′*S*)-2-hydroxyastaxanthin, (2*R*,3*S*,2′*R*,3′*R*)-2,2′-dihydroxyadonixanthin [(2*R*,3*S*,2′*R*,3′*R*)-2,3,2′,3′-tetrahydroxy-*β*,*β*-caroten-4-one], (2*R*,3*S*,3′*R*)-2-hydroxyadonixanthin, and (3*S*,2′*R*,3′*R*)-erythroxanthin, in addition to (3*S*,3′*S*)-astaxanthin and (3*S*,3′*R*)-adonixanthin. However, the production of 2,2′-dihydroxyastaxantin was at a small scale, and 2,2′-dihydroxyadonirubin was not detected in the SD212 strain. Liu et al. reported that a marine bacterium *Brevundimonas scallop* mainly produced 2,2′-dihydroxyastaxantin and astaxanthin [38].

**Figure 4:**
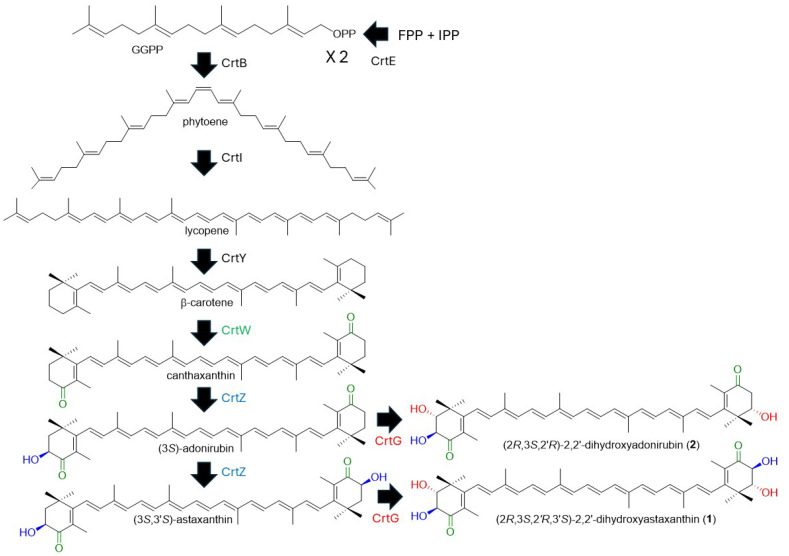
Feasible biosynthetic pathway of (2*R*,3*S*,2′ *R*,3′*S*)-2,2′-dihydroxyastaxanthin (**1**) and (2*R*,3*S*,3′*R*)-2-hydroxyadonixanthin (**2**). IPP, isopentenyl diphosphate; FPP, farnesyl diphosphate; and GGPP, geranylgeranyl diphosphate.

We achieved the dominant production of the 2,2′-dihydroxy derivatives of astaxanthin and its intermediate adonirubin in *E. coli* cells. This result should pave the way for the heterologous production of these carotenoids with reasonable hosts. Three genes, *crtZ*, *crtW*, and *crtG*, are needed to metabolize β-carotene to 2,2′-dihydroxyastaxanthin. We here adopted the *Pantoea ananatis crtZ* (β-carotene/canthaxanthin 3,3′-hydroxylase) gene, the *Paracoccus* sp. strain N81106 *crtW* (β-carotene/zeaxanthin 4,4′-ketolase) gene, and the *Brevundimonas* sp. strain SD212 *crtG* (β,β-carotenoid 2,2′-hydroxylase) gene, since these gene-encoded CrtW, CrtZ, and CrtG enzymes can exert broad substrate preferences for the carotenoid β-ionone ring (β-end group). The *Brevundimonas* sp. SD212 CrtG was reported to have almost no catalytic activity on β-carotene and catalytic activity on zeaxanthin, canthaxanthin, and astaxanthin [11]. The appropriate expression of all the three genes in the β-carotene-synthesizing *E. coli* should result in the dominant productions of 2,2′-dihydroxyastaxanthin. Its feasible biosynthetic pathway is shown in Figure 4. 2,2′-Dihydroxyadonirubin (**2**), that was dominantly produced in the recombinant *E. coli* cells, was not observed in the original SD212 strain [12]. We utilized the *crtW* and *crtZ* genes not from the *Brevundimonas* SD212 strain. Thus, such a difference may have occurred.

The carotenoid biosynthesis sections of the Enzyme Commission (EC) classification and pathway databases such as KEGG appear to contain several inconsistencies and potential misclassifications. Here, we describe the historical establishment of the *crtZ*, *crtW*, and *crtG* genes.

The designation “*crt*” for bacterial carotenoid biosynthesis genes originated from studies on photosynthetic bacteria such as *Rhodobacter* and *Rhodospirillum* species (class *Alphaproteobacteria*), which produce acyclic C40 carotenoids [42,43]. The nucleotide sequence of the *Rhodobacter capsulatus crt* gene cluster was published in 1989 [43], providing the first comprehensive view of carotenogenic gene organization. Prior to this, gene structures had largely been inferred through mutational analyses, while the precise enzymatic functions often remained unclear.

Misawa et al. (1990) subsequently reported the nucleotide sequence of the carotenoid biosynthesis gene cluster from *Erwinia uredovora* strain 20D3, later reclassified as *Pantoea ananatis* (class *Gammaproteobacteria*) [44]. Importantly, various combinations of genes within this cluster were heterologously expressed in *E. coli*, and the resulting carotenoid pigments were chemically analyzed to determine their chemical structures. This experimental strategy enabled the direct identification of substrates and products corresponding to each *crt* gene product, thereby establishing reaction-based functional assignments for individual enzymes. Such a reaction-defined approach represented a decisive methodological advance in the functional characterization of carotenoid biosynthetic genes following nucleotide sequence determination.

An initially uncharacterized open reading frame (ORF) within the *Pantoea ananatis crt* gene cluster, designated *crtZ*, was shown to convert β-carotene into (3*R*,3′*R*)-zeaxanthin, demonstrating that *crtZ* encodes a β-carotene 3,3′-hydroxylase [44]. Several years later, *crtZ* genes from *P. ananatis*, as well as from the astaxanthin-producing marine bacteria *Agrobacterium aurantiacum* and *Alcaligenes* sp. strain PC-1, subsequently reclassified as *Paracoccus* species (class *Alphaproteobacteria*), were further shown to convert canthaxanthin into (3*S*,3′*S*)-astaxanthin via adonirubin (phoenicoxanthin). These findings established CrtZ enzymes in members of the phylum *Pseudomonadota* (formerly *Proteobacteria*) as β-carotene/canthaxanthin 3,3′-hydroxylases [45,46].

Similarly, initially uncharacterized ORFs designated *crtW* within the *crt* gene clusters of the astaxanthin-producing marine bacteria *A. aurantiacum* and *Alcaligenes* sp. strain PC-1 (later renamed *Paracoccus* sp. strain N81106 and *Paracoccus* sp. strain PC-1, respectively) were shown to convert β-carotene into canthaxanthin via echinenone, demonstrating that *crtW* encodes a β-carotene 4,4′-ketolase [47]. These *crtW* genes were further shown to convert (3*R*,3′*R*)-zeaxanthin into (3*S*,3′*S*)-astaxanthin via adonixanthin (4-ketozeaxanthin). These findings established CrtW enzymes in members of the phylum *Pseudomonadota* as β-carotene/zeaxanthin 4,4′-ketolases [45,46].

Fraser et al. (1997) revealed through in vitro analyses that CrtZ and CrtW are strictly oxygen-dependent enzymes requiring Fe^2+^ and 2-oxoglutarate as cofactors [46], findings that were supported by cultivation studies [48]. These results demonstrated that both enzymes belong to the family of Fe^2+^/2-oxoglutarate-dependent oxygenases.

Similarly, an initially uncharacterized ORF designated *crtG* within the *crt* gene cluster of the 2,2′-dihydroxyastaxanthin-producing marine bacterium *Brevundimonas* sp. strain SD212 was shown to convert (3*R*,3′*R*)-zeaxanthin and canthaxanthin into (2*R*,2′*R*)-2,2′-dihydroxyzeaxanthin (nostoxanthin) via (2*R*)-2-hydroxyzeaxanthin (caloxanthin) and (2*R*,2′*R*)-2,2′-dihydroxycanthaxanthin via (2*R*)-2-hydroxycanthaxanthin, respectively [11]. This *crtG* was also found to generate 2-hydroxyastaxanthin from astaxanthin [11]. The present study clarified that the *crtG* can convert astaxanthin into 2,2′-dihydroxyastaxanthin via 2-hydroxyastaxathin. The *crtG* gene from another *Brevundimonas* species, *Brevundimonas aurantiaca*, was shown to have the same function as strain SD212 *crtG* [34]. These findings established CrtG enzymes in members of the phylum *Pseudomonadota* as zeaxanthin/canthaxanthin/astaxanthin 2,2′-hydroxylase.

Collectively, these studies firmly anchored the *crtZ*, *crtW*, and *crtG* designations to reaction-defined activities—β-carotene/canthaxanthin 3,3′-hydroxylation, β-carotene/zeaxanthin 4,4′-ketolation, and zeaxanthin/canthaxanthin/astaxanthin 2,2′-hydroxylation, respectively—within the phylum *Pseudomonadota.*

The heterologous identification and functional validation of *crtZ* and *crtW* in the early 1990s represented more than a clarification of gene function; it provided a practical framework for reconstructing carotenoid pathways in non-native hosts. The ability to introduce defined hydroxylation and ketolation steps, together with β-carotene biosynthesis genes, into *E. coli* and other suitable organisms (including bacteria and yeasts), enabled the stepwise production of high-value ketocarotenoids such as astaxanthin, thereby establishing a gene-based modular strategy for efficient carotenoid biosynthesis.

Subsequently, a large number of studies have employed microbial hosts for heterologous astaxanthin production, e.g., including *E. coli* [49,50,51,52], *Corynebacterium glutamicum* [53], a marine photosynthetic bacterium [54], a methanotrophic bacterium [55], a cyanobacterium [56], and the yeasts *Candida utilis* [57], *Saccharomyces cerevisiae* [58], and *Yarrowia lipolytica* [59,60].

Thus, the early heterologous characterization of *crtZ* and *crtW* not only anchored nomenclature in biochemical evidence but also laid conceptual and technical foundations for modern carotenoid metabolic engineering followed by synthetic biology on carotenoids. In this sense, the *crtZ/crtW*-based heterologous system can be regarded as one of the earliest practical demonstrations of gene-based modular pathway design in carotenoid biosynthesis.

The XLogP [61,62] (an index of lipophilicity) values of **1**, **2**, and astaxanthin were calculated using Anaconda Python (RDkit), and these were 6.85, 7.88, and 8.91, respectively. These values indicate that the *n*-octanol/water partition ratios of **1** and **2** are more than an order of magnitude lower than that of astaxanthin, demonstrating their substantially higher polarity and predicting higher aqueous solubility compared with astaxanthin.

The reduction in lipophilicity is expected to influence several pharmacokinetic and pharmacodynamic properties relevant to its biological performance. Highly lipophilic carotenoids often suffer from limited dispersion in aqueous biological environments, resulting in poor gastrointestinal dissolution, restricted membrane transport, and suboptimal systemic bioavailability [63,64,65]. By contrast, a moderate decrease in hydrophobicity can enhance colloidal stability, facilitate incorporation into mixed micelles, and improve interaction with lipid transporters, thereby promoting more efficient intestinal absorption [64,66].

In addition, carotenoid antioxidant activity is strongly affected by their localization and orientation within lipid bilayers. Astaxanthin’s rigid, highly hydrophobic polyene chain typically anchors deeply within membranes, which contributes to its potent singlet oxygen-quenching capacity but may also limit its mobility and accessibility to reactive species in heterogeneous biological compartments [67]. The lower XlogPs of **1** and **2** suggest a more favourable balance between membrane affinity and aqueous dispersibility, potentially enabling more dynamic partitioning between lipid and aqueous phases. Such physicochemical behaviour may enhance its ability to intercept reactive oxygen species at membrane interfaces, where oxidative processes are most prevalent [67].

Enhanced bioavailability, in turn, could potentiate downstream physiological effects, including anti-inflammatory, cytoprotective, and mitochondrial-supporting activities that are well documented for astaxanthin [63]. Although empirical validation is required, the physicochemical profile of **1** and **2** provides a strong rationale for expecting superior biological performance relative to native astaxanthin.

The singlet oxygen (^1^O_2_)-quenching activities of compounds **1** (IC_50_ 4.3 μM), **2** (IC_50_ 8.3 μM), and astaxanthin (IC_50_ 1.7 μM) were systematically evaluated and compared with that of canthaxanthin (IC_50_ 2.3 μM), following the previously established methodology [41]. As shown in Table 1, both **1** and **2** exhibited ^1^O_2_-quenching activities that were essentially comparable to that of astaxanthin, despite their structural modifications. This finding is particularly noteworthy because alterations to the polyene backbone or terminal ionone rings of carotenoids often lead to substantial reductions in photochemical reactivity [4]. The preservation of high quenching efficiency in **1** and **2** therefore indicates that the modifications introduced in **1** and **2** do not compromise the conjugated π-electron system responsible for energy transfer to ^1^O_2_.

A closer examination of the structure–activity relationship suggests that hydroxylation at the C-3 position in the 4-keto-β-ionone ring enhances ^1^O_2_-quenching activity, consistent with the well-established role of 3-hydroxy groups in stabilizing excited-state intermediates [68,69]. In contrast, hydroxylation at C-2 in the β-ionone ring appears to exert a modest attenuating effect; however, the underlying mechanism for this attenuation remains unclear. Nevertheless, the overall activities of **1** and **2** remains within the same functional range as astaxanthin, underscoring the robustness of the carotenoid scaffold toward selective hydroxylation. These results highlight that **1** and **2** retain the key photoprotective functionality characteristic of astaxanthin while exhibiting distinct structural features that may be advantageous for further optimization. The maintenance of strong ^1^O_2_-quenching activity, despite modifications at C-2 and C-3, positions these analogues as promising candidates for applications requiring potent singlet oxygen suppression, such as photoprotection, oxidative stress mitigation, and functional food or nutraceutical development.

A previous study reported that both **1** and astaxanthin exhibited DPPH radical scavenging activity at concentrations of 5–20 μg/mL [38]. To directly compare these findings under identical experimental conditions, we re-evaluated **1**, **2**, and astaxanthin using the same assay parameters. However, none of the compounds demonstrated measurable scavenging activity within this concentration range in our hands.

We are confident that our groups have taken pioneering steps in expanding a heterologous expression platform capable of producing target carotenoids that are otherwise difficult to obtain from natural sources. In the present study, we introduce a new approach to improve the water solubility, bioavailability, and pharmacological activity of astaxanthin through employing not PEGylation or nanoencapsulation of astaxanthin but structurally related, non-glycosylated astaxanthin derivatives.

In summary, we successfully produced two hydrophilic astaxanthin-related carotenoids [a rare carotenoid (2*R*,3*S*,2′*R*,3′*S*)-2, 2′-dihydroxyastaxanthin (**1**) and a new carotenoid (2*R*,3*S*,2′*R*)-2,2′-dihydroxyadonirubin (**2**)] using recombinant *E. coli* engineered with carotenoid biosynthetic genes. Both compounds exhibited strong singlet oxygen-quenching activity, only slightly lower than that of astaxanthin, and higher water solubility than astaxanthin. We will conduct a series of biological activity assays to further evaluate the potential of **1** and **2** as promising antioxidant candidates.

## Figures and Tables

**Table 1:** ^1^O_2_-quenching activities (IC_50_ (μM)) of the carotenoids.

Carotenoid	IC50 (μM)
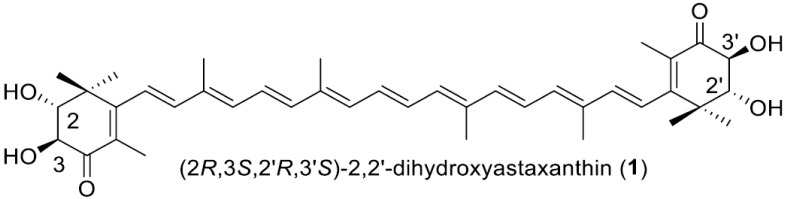	4.3 ± 1.18
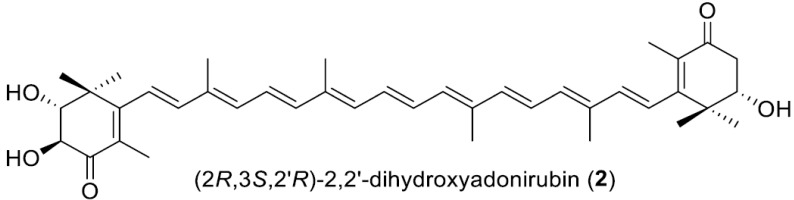	8.3 ± 0.43
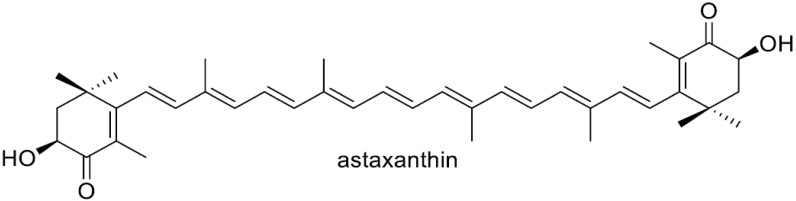	1.7 ± 0.14
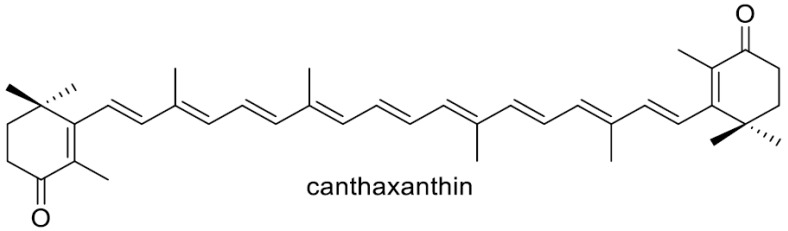	2.3 ± 0.21 ^a)^

^a)^ Cited from our previous data [41].

## Data Availability

All data generated in this study are included in the article and its Supplementary Materials. Further inquiries can be directed to the corresponding author.

## Supplementary Materials

The following supporting information can be downloaded at: https://www.mdpi.com/article/10.3390/antiox15030327/s1, Figure S1: ^1^H NMR spectrum of (2*R*,3*S*,2′*R*,3′*S*)-2,2′-dihydroxyastaxanthhin (**1**) in CDCl_3_; Figure S2: ^13^C NMR spectrum of (2*R*,3*S*,2′*R*,3′*S*)-2,2′-dihydroxyastaxanthhin (**1**) in CDCl_3_; Figure S3: ^1^H NMR spectrum of (2*R*,3*S*,2′*R*)-2,2′-dihydroxyadnirubin (**2**) in CDCl_3_; Figure S4: ^13^C NMR spectrum of (2*R*,3*S*,2′*R*)-2,2′-dihydroxyadnirubin (**2**) in CDCl_3_; Figure S5: DPPH radical scavenging activities of (2*R*,3*S*,2*R*,3′*S*)-2,2′-dihydroxyastaxanthin (**1**), (2*R*,3*S*,2′*R*)-2,2′-dihydroxyadonirubin (**2**), and astaxanthin.
