# Supplementary material for: Heterologous Production of 2,2′-Dihydroxy Derivatives of Astaxanthin and Adonirubin in Escherichia coli and Evaluation of Their Antioxidant Activity
Source: Antioxidants (Basel). 2026 Mar 5;15(3):327. doi: 10.3390/antiox15030327 (PMC13023446; doi:10.3390/antiox15030327)
Supplement: Supplementary file 1 [file antioxidants-15-00327-s001.zip › antioxidants-4126064-supplementary.pptx]

## Slide 1
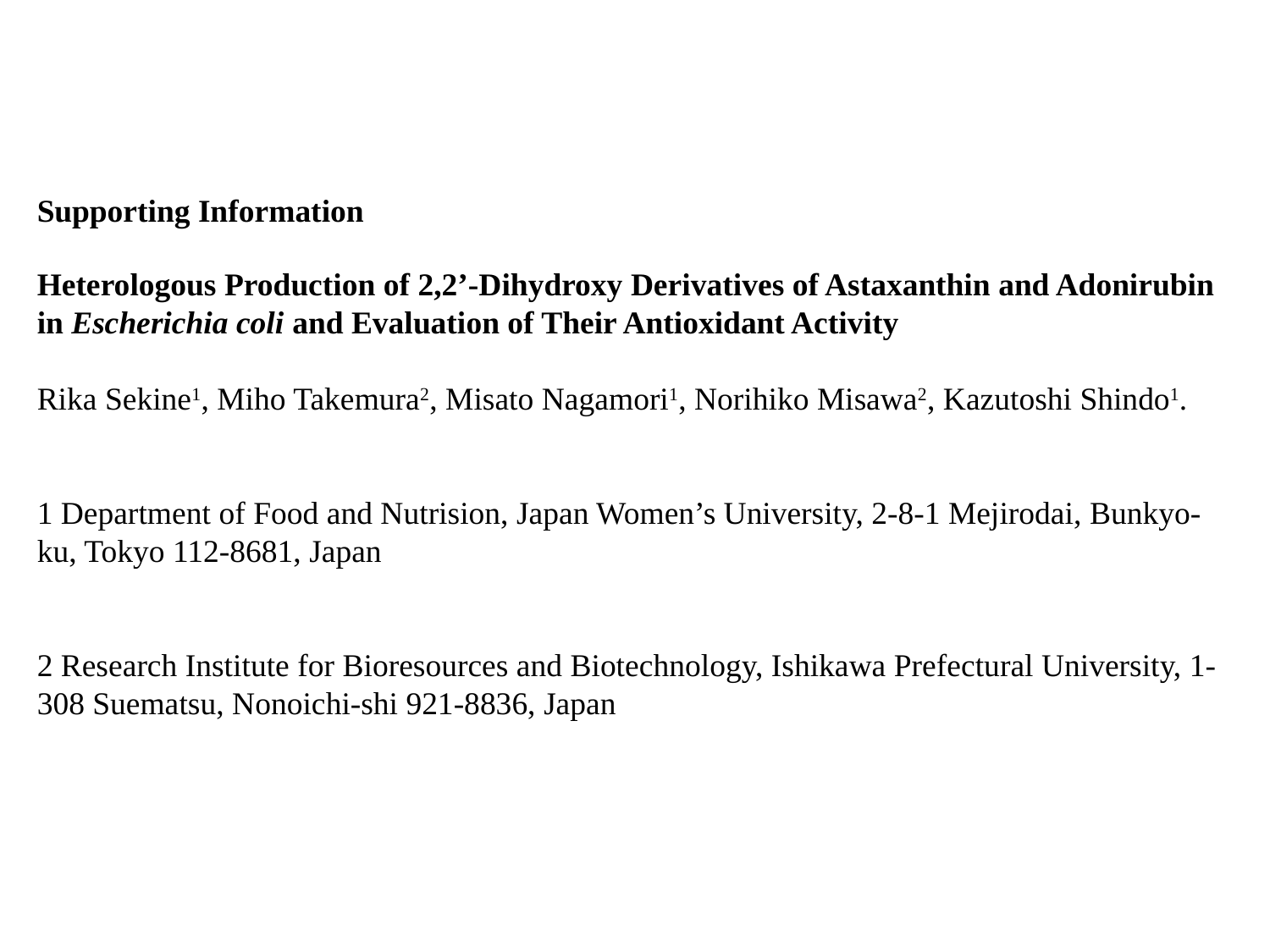

Supporting Information
Heterologous Production of 2,2’-Dihydroxy Derivatives of Astaxanthin and Adonirubin in Escherichia coli and Evaluation of Their Antioxidant Activity
Rika Sekine1, Miho Takemura2, Misato Nagamori1, Norihiko Misawa2, Kazutoshi Shindo1.
1 Department of Food and Nutrision, Japan Women’s University, 2-8-1 Mejirodai, Bunkyo-ku, Tokyo 112-8681, Japan
2 Research Institute for Bioresources and Biotechnology, Ishikawa Prefectural University, 1-308 Suematsu, Nonoichi-shi 921-8836, Japan

## Slide 2
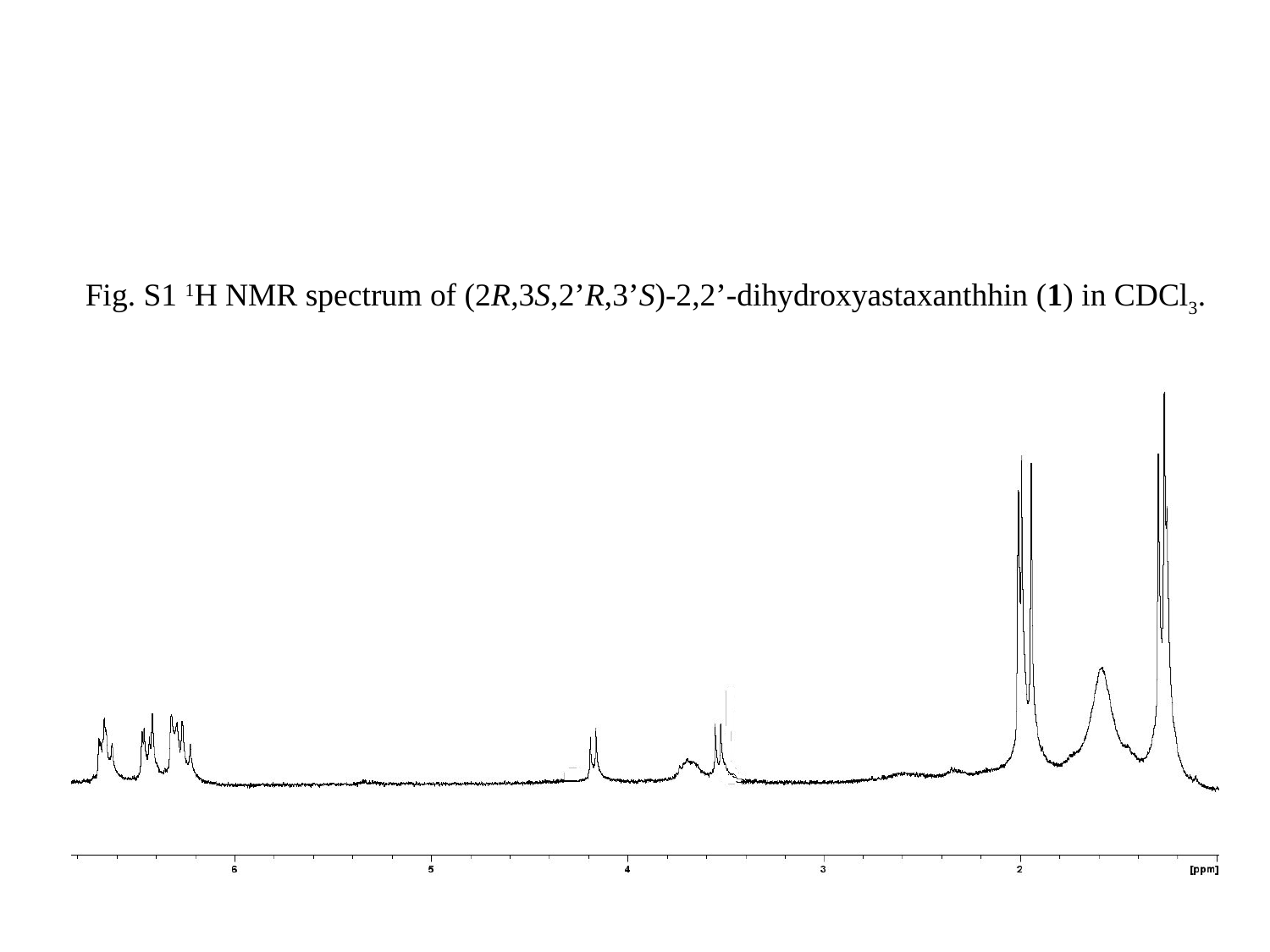

Fig. S1 1H NMR spectrum of (2R,3S,2’R,3’S)-2,2’-dihydroxyastaxanthhin (1) in CDCl3.

## Slide 3
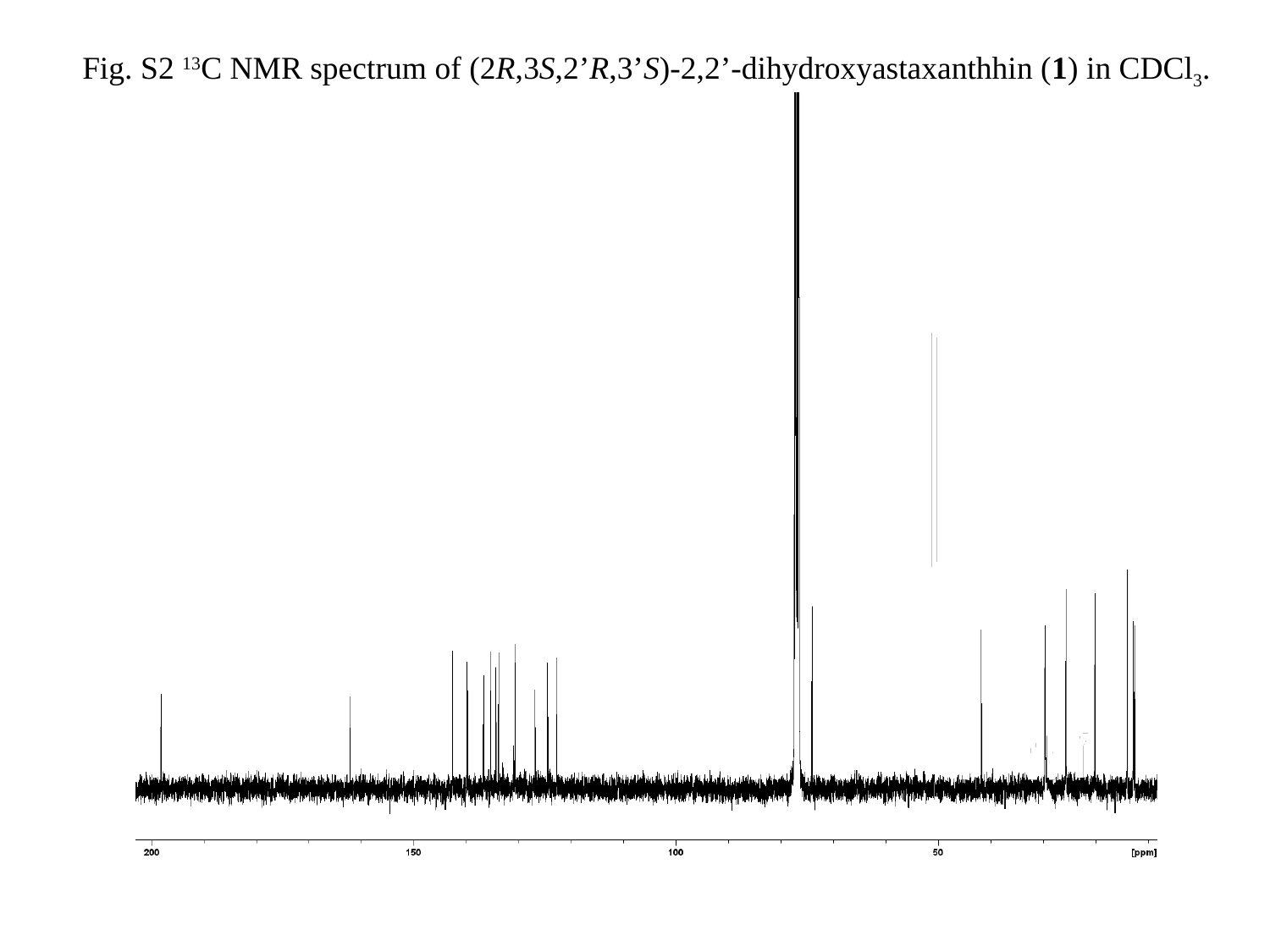

Fig. S2 13C NMR spectrum of (2R,3S,2’R,3’S)-2,2’-dihydroxyastaxanthhin (1) in CDCl3.

## Slide 4
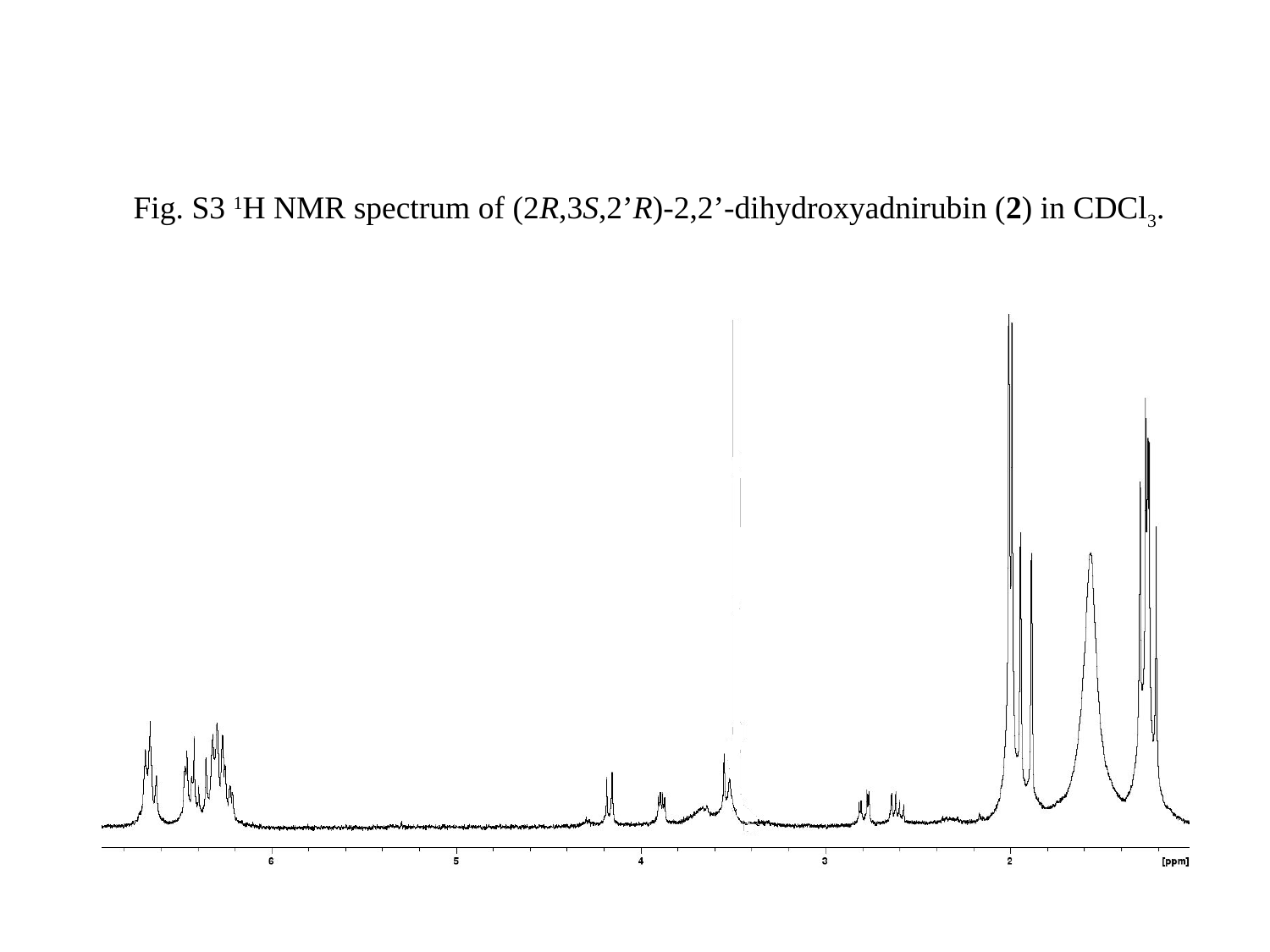

Fig. S3 1H NMR spectrum of (2R,3S,2’R)-2,2’-dihydroxyadnirubin (2) in CDCl3.

## Slide 5
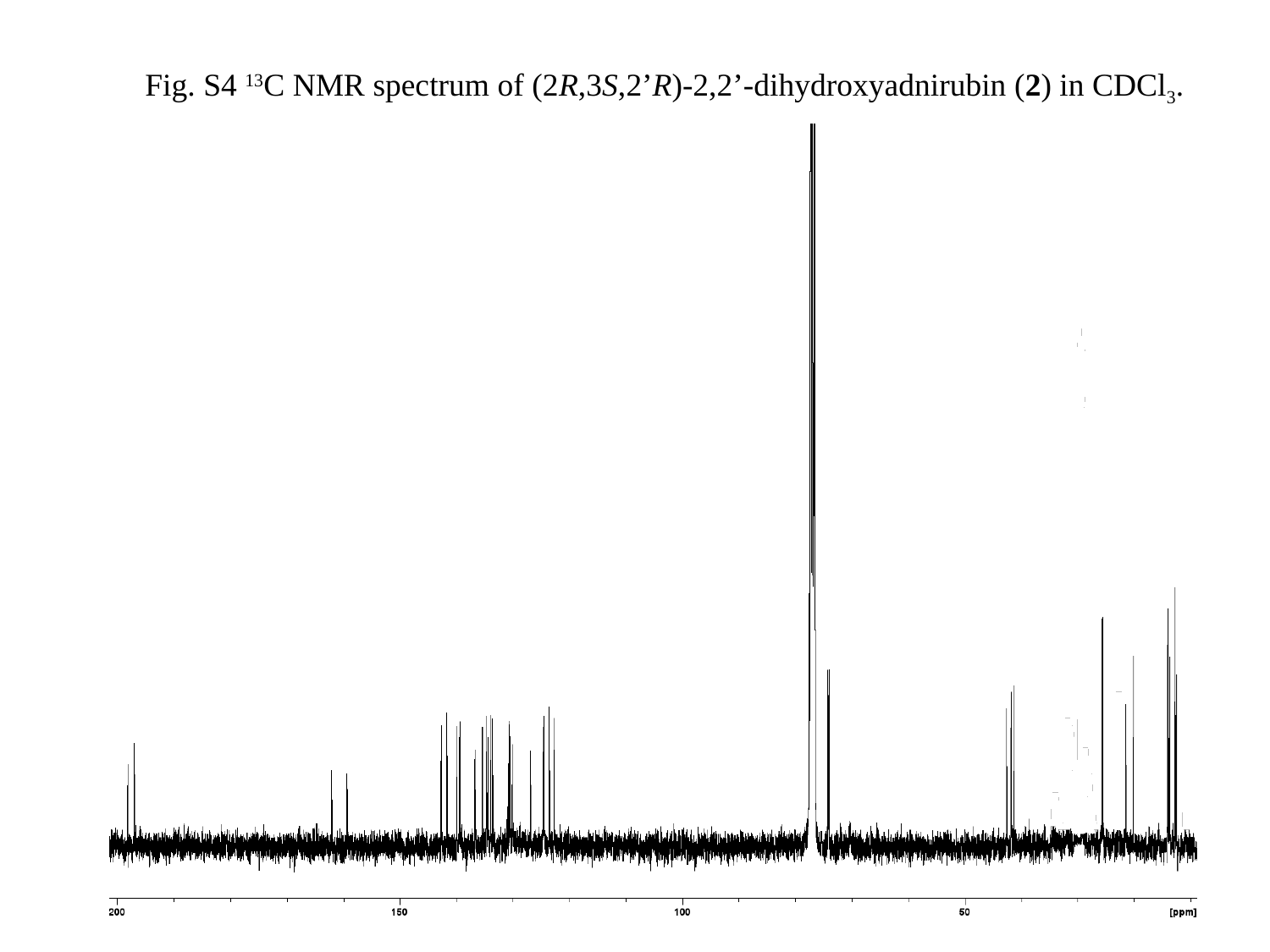

Fig. S4 13C NMR spectrum of (2R,3S,2’R)-2,2’-dihydroxyadnirubin (2) in CDCl3.

## Slide 6
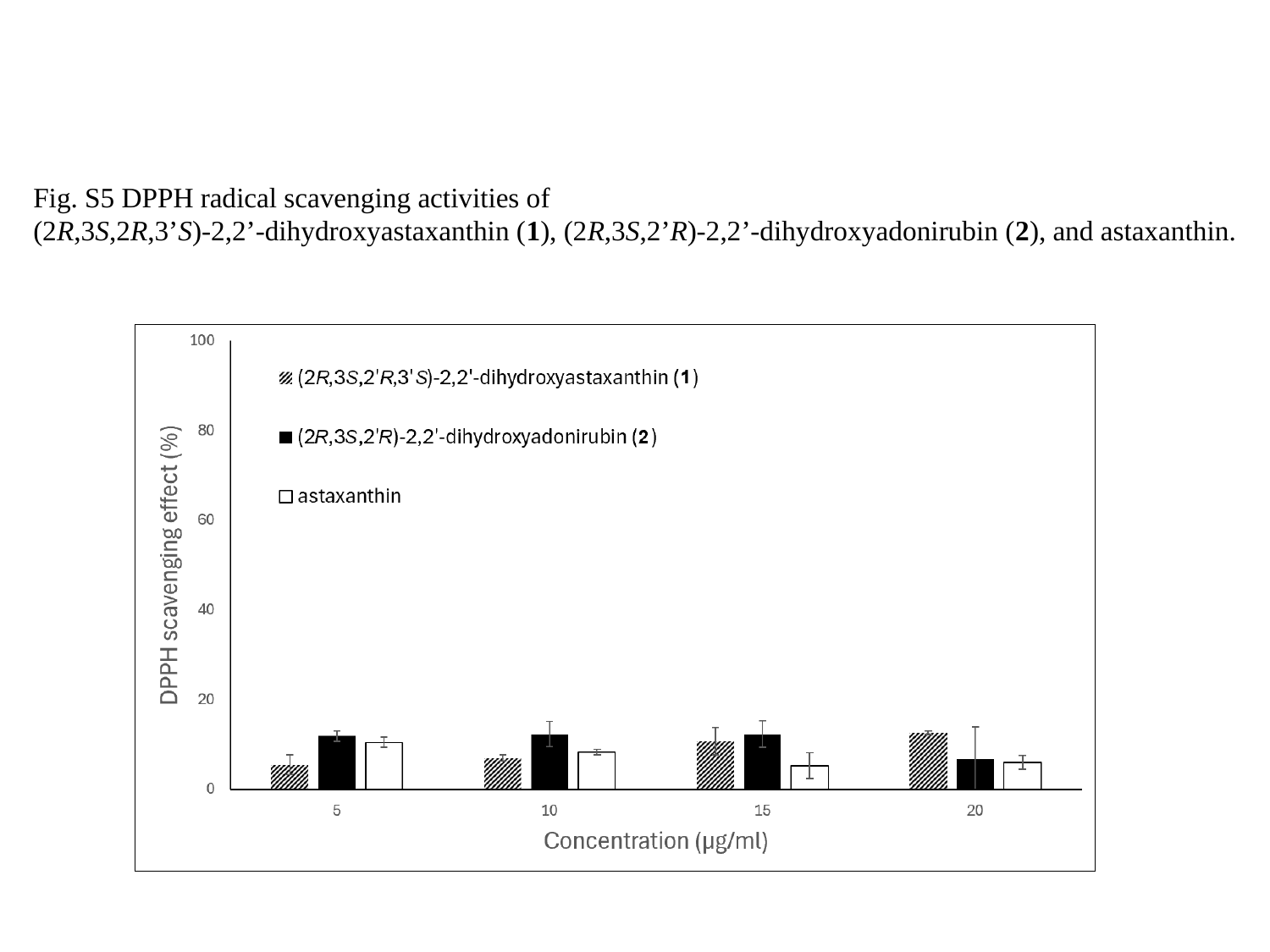

Fig. S5 DPPH radical scavenging activities of
(2R,3S,2R,3’S)-2,2’-dihydroxyastaxanthin (1), (2R,3S,2’R)-2,2’-dihydroxyadonirubin (2), and astaxanthin.
